# Identification and Validation of Immune-Related Gene for Predicting Prognosis and Therapeutic Response in Ovarian Cancer

**DOI:** 10.3389/fimmu.2021.763791

**Published:** 2021-11-22

**Authors:** Zhao-Cong Zhang, Jun-Nan Guo, Ning Zhang, Zhi-Qiang Wang, Ge Lou, Bin-Bin Cui, Chang Yang

**Affiliations:** ^1^ Department of Gynecology Oncology, Harbin Medical University Cancer Hospital, Harbin, China; ^2^ Department of Colorectal Surgery, Harbin Medical University Cancer Hospital, Harbin, China

**Keywords:** ovarian cancer, immune-related genes (IRGs), prognosis, tumor immune microenvironment, immune checkpoint inhibitors (ICI)

## Abstract

Ovarian cancer (OC) is a devastating malignancy with a poor prognosis. The complex tumor immune microenvironment results in only a small number of patients benefiting from immunotherapy. To explore the different factors that lead to immune invasion and determine prognosis and response to immune checkpoint inhibitors (ICIs), we established a prognostic risk scoring model (PRSM) with differential expression of immune-related genes (IRGs) to identify key prognostic IRGs. Patients were divided into high-risk and low-risk groups according to their immune and stromal scores. We used a bioinformatics method to identify four key IRGs that had differences in expression between the two groups and affected prognosis. We evaluated the sensitivity of treatment from three aspects, namely chemotherapy, targeted inhibitors (TIs), and immunotherapy, to evaluate the value of prediction models and key prognostic IRGs in the clinical treatment of OC. Univariate and multivariate Cox regression analyses revealed that these four key IRGs were independent prognostic factors of overall survival in OC patients. In the high-risk group comprising four genes, macrophage M0 cells, macrophage M2 cells, and regulatory T cells, observed to be associated with poor overall survival in our study, were higher. The high-risk group had a high immunophenoscore, indicating a better response to ICIs. Taken together, we constructed a PRSM and identified four key prognostic IRGs for predicting survival and response to ICIs. Finally, the expression of these key genes in OC was evaluated using RT-qPCR. Thus, these genes provide a novel predictive biomarker for immunotherapy and immunomodulation.

## Introduction

Ovarian cancer (OC) is one of the most lethal gynecological malignancies. Because the early symptoms are not obvious and progress is rapid, it is usually diagnosed during the late stages (1). At present, the clinical treatment of OC is based on surgery and chemotherapy; however, they do not substantially improve survival (2). Therefore, immunotherapy for OC has attracted widespread attention. A consensus that OC is an immunogenic tumor has been reached among researchers (3). The combined application of OC immunotherapy and traditional treatment methods can improve the treatment effect (4, 5), but the prognosis is important differences. Therefore, further insights into the mechanisms underlying these differences are essential for the discovery of tumor prognostic markers.

The tumor microenvironment plays a vital role in tumor occurrence and development. Among the slew of micro-environment factors, the heterogeneity of the immune tumor microenvironment affects the treatment effect of patients and is a potential obstacle to the development of personalized immunotherapy (6, 7). Thus, looking for differentially expressed genes (DEGs) in the tumor immune microenvironment, along with evaluating their functions, is expected to result in new immune checkpoints.

Herein, we investigated the ability to predict disease prognosis based on immune-related genes (IRGs) differentially expressed in the tumor microenvironment. Differentially expressed gene data for patients with OC were downloaded from The Cancer Genome Atlas (TCGA) database. For module detection, we applied a weighted gene co-expression network analysis (WGCNA) to select the module gene with the highest correlation with the immune score in order to construct immune-related gene pairs (IRGPs). Furthermore, a prognostic risk scoring model (PRSM) was created using the IRGPs. We used the Gene Expression Omnibus (GEO) database to verify the PRSM, which was used to calculate the patient’s risk score (RS), and then divided the patients into low-risk and high-risk groups (LRG and HRG, respectively). We then identified key prognostic (IRGs). Finally, we downloaded response data to chemotherapy drugs from CellMiner™ (https://discover.nci.nih.gov/cellminer/) to analyze the relationship between IRGs and drug resistance.

## Materials and Methods

### OC Samples Data Collection and Processing

In our study, we used tissue samples from different high-throughput platforms, namely TCGA (https://portal.gdc.cancer.gov/) and GEO (http://www.ncbi.nlm.nih.gov/geo/). We gathered 664 OC high-throughput gene datasets containing 379 samples from TCGA and 285 samples from GEO (GSE9891). Only 505 patients with complete information were included in the analysis, comprising 375 OC samples from TCGA and 130 OC samples from GEO (GSE103479). We converted the gene ID to the matching gene symbols according to the annotation package corresponding to each dataset. TCGA data were chosen as the model group and GEO data were used as the verification group. In the analysis, we excluded RNA that could not be detected in >10% of the samples.

### Preliminary Screening of IRGs

The R package “ESTIMATE” (8) is an algorithm based on ssGSEA, which is used to evaluate the immune infiltration in TCGA samples. The expression matrix of each tumor sample was scored using two related gene sets: stromal and immune. Then, the R package “maxstat” (9) was employed to calculate the cut-off values of the immune and matrix scores. Subsequently, all samples were divided into the high/low immune and stromal score groups. The R package “survcomp” (10) was used for visualization, and a Kaplan-Meier survival curve was obtained. Moreover, the same R package was used to compare survival differences between the two groups based on the log-rank test. The R package “Limma” (11) was used to analyze DEGs (|log2foldchange| > 0.5, *p*-adj < 0.05). Subsequently, we took the intersection between up- and down-regulated DEGs, and screened out the IRGs in OC, displaying them using a Venn diagram.

### Establishment of Co-Expression Algorithm of IRGs

A WGCNA (12) was used to identify consensus gene modules from IRGs, analyze modules, and calculate the correlation of results using “ESTIMATE”. First, we constructed an adjacency matrix (AM) of paired genes using a power function. An appropriate power index was selected to increase the similarity of the matrix and build a scale-free co-expression network. The AM was then converted into a topological overlap matrix (TOM). Based on the TOM dissimilarity measurements, we performed an average linkage hierarchical cluster analysis. The correlation coefficient (CC) was determined between module eigengene and stromal and immune scores. Gene significance (GS) was defined as the mediated *p*-value of each gene (GS = lg*P*) in the linear regression between gene expression and scores. Finally, a gene clustering tree was constructed based on the correlation between the expression levels of genes and the gene module.

### Further Screening of IRGs and Construction of IRGPs

To further identify IRGs and construct immune gene pairs, we selected the module with the highest CC with the immune score and calculated the GS and module membership (MM) in this module. MM is a measure of the connectivity between genes and modules. The threshold was defined as a cor. gene MM > 0.7 and cor. gene GS > 0.7. We constructed gene pairs for the selected IRGs to eliminate sequencing errors between different platforms and samples. Specifically, the expression levels of any two genes were compared in each sample. If the former was greater than the latter, it was recorded as 1, and vice versa. After removing the IRGPs with minimal expression and uneven distribution (MAD = 0), univariate Cox proportional hazards regression analysis was performed on the remaining IRGPs in the model group. The IRGPs with *p* < 0.05 in Cox regression were retained for Lasso-Cox proportional hazards regression with 1000 simulations using the R package “glmnet” (13). The receiver operating characteristics (ROC) for 5 years was drawn using R package “survival ROC” (14) and the area under the curve was determined. The best cut-off points were marked on the ROC curves. Finally, a predictive model was applied to the validation group. All patients were classified into either the HRG or LRG based on the optimal cut-off of the RS.

### Validation of the Predictive Model

Prognostic analysis was performed on high- and low-risk patients and validation groups using the log-rank test to verify the accuracy and consistency of the PRSM. Then, in the model group with complete clinical information, the risk score was combined with other clinical factors to perform single-factor and multi-factor Cox regression analysis to further verify the independent influence of the RS.

### Immune Infiltration in the HRG and LRG

To elucidate differences in immune cell infiltration between the HRG and LRG, we adopted another algorithm to estimate the relative infiltration abundance of 22 immune cells in different samples by using the R package “CIBERSORT” (15). Then, we reserved the samples with *p* < 0.05, estimated using “CIBERSORT”, and performed a differential analysis of the content of various immune cells in the HRG and LRG using a Wilcoxon rank-sum test.

### Functional Enrichment Analysis

For the purpose of studying the biological functions of differential IRGs and genes in the PRSM, we employed the bioconductor package “fgsea” (16) to perform Gene Ontology- (GO)- and Kyoto Encyclopedia of Genes and Genomes- (KEGG)- related gene set enrichment analysis (GSEA) with 10,000 permutations. To compare genes between the HRG and LRG, we performed log2 multiple conversions and sorted the ratio of gene expression. The threshold values were set to *p* < 0.05.

### Identification of Key Prognostic IRGs

To further screen the key prognostic IRGs, we performed protein interaction network analysis using STRING (https://www.string-db.org) on the IRGPs. We selected the top 30 genes in the network and the genes in the model to intersect and analyze the prognosis of the intersection genes. After obtaining the key prognostic IRGs, we used the DisNor database (https://disnor.uniroma2.it/) to analyze their upstream and downstream related proteins and mode of action. DisNor is a disease-focused resource that uses the causal interaction information annotated in SIGNOR and the protein interaction data in Mentha to generate and explore protein interaction networks linking disease genes.

### Validation of DEGs Using RT-qPCR

The expression of these key prognostic IRGs in fresh frozen OC tissue samples was determined using RT-qPCR. Fresh ovarian cancer tissues and normal ovarian tissues were obtained from Harbin Medical University Cancer Hospital. Trizol reagent (Ambion, Shanghai, China) was added to OC tissues, and total RNA was extracted according to the manufacturer’s instructions. After elution with RNase-free water, RNA was stored at – 80°C until further analysis. RNA quality was evaluated using a spectrophotometer (Eppendorf), and then reverse-transcribed into cDNA using a reverse transcription kit (Vazyme, Nanjing, China). RT-qPCR was performed using SYBR Green PCR kit. The RT-qPCR primers for CD163, Toll-like receptor 4 (TLR4), Bruton’s tyrosine kinase (BTK), and C3AR1 were designed using the Prime Bank website (17). GAPDH was used as an internal control. PCR cycling conditions were as follows: 95°C for 2 min, followed by 94°C for 20 s, 58°C for 20 s, and 72°C for 30 s for 40 cycles. For RT-qPCR experiments, all samples were prepared in triplicate. Expression of these genes was calculated using the comparative cycle threshold (2^−ΔΔCt^) method.

### A Sensitivity Analysis of Different Treatment Modalities

To evaluate the value of prediction models and key prognostic IRGs in the clinical treatment of OC, we analyzed the treatment sensitivity from three aspects: chemotherapy drugs, targeted inhibitors (TIs), and immunotherapy.

First, we calculated the 50% reduction growth (IC50) concentration caused by TIs using the R package “pRRophetic” (18), including AKT, Hedgehog (HH), VEGFR, and JNK/STAT inhibitors. The Wilcoxon rank- sum test was used to compare the difference in IC50 between the different risk groups. In addition, we downloaded gene expression data and response data to chemotherapy drugs from CellMiner™ (https://discover.nci.nih.gov/cellminer/) from the same batch. Drugs that were clinically tested and not approved by the FDA were excluded. We then extracted key prognostic IRGs from gene expression data and analyzed the correlation between their expression and drug sensitivity.

Immunogenicity is determined by a variety of IRGs, including genes related to effector cells, immunosuppressive cells, major histocompatibility complex molecules, and immune regulatory factors. Using machine learning, the immune-phenotyping score (IPS) can unbiasedly assess and quantify immunogenicity. To evaluate the effect of immunotherapy, we downloaded the IPS of patients with OC from the TCIA database (https://tcia.at/) and compared the IPS between the HRG and LRG in different immunotherapy decisions. In addition, we analyzed the differences in the expression of seven important immuno-suppressive checkpoint genes in the HRG and LRG.

## Results

### Grouping of DEGs Based on Immune and Stromal Scores

**Figure 1:**
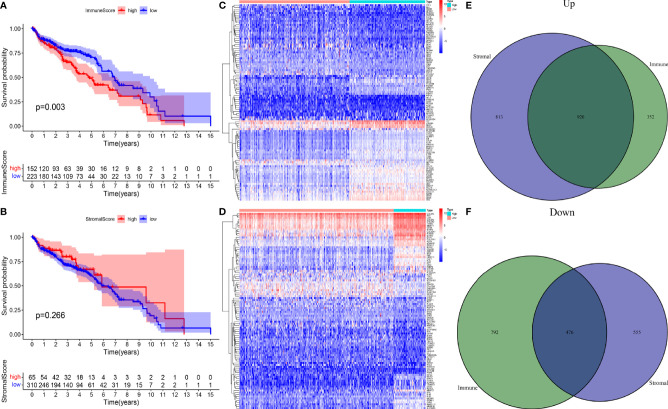
**(A)** A survival curve based on immune score for patients in the HRG and LRG. **(B)** A survival curve based on stromal score for patients in the HRG and LRG. **(C)** Heatmap plots of DEGs in immune score of OC. **(D)** Heatmap plots of DEGs in stromal score of OC. **(E)** Venn diagram depicting the number of upregulated DEGs based on two scores. **(F)** Venn diagram depicting the number of downregulated DEG based on two scores.

To study the differentially expressed IRGs of OC, TCGA data were filtered, grouped, normalized, and differentially expressed. Through these processes, 1,408 DEGs were screened out, which were divided into HRG and LRG according to their immune and stromal scores. The Kaplan- Meier survival curve, based on immune score, was plotted for patients in the HRG and LRG, and the results showed that the overall survival (OS) of the HRG was significantly lower than that of the LRG (*p* = 0.003) (**Figure 1A**). However, there was no statistically significant difference in OS between the HRG and LRG based on the stromal score (*p* = 0.266) (**Figure 1B**). Subsequently, we analyzed the DEGs in different groups of samples separately based on the two scores through the R package “Limma” and divided them into high expression and low expression (**Figures 1C, D**). The Venn diagrams (**Figures 1E, F**) demonstrate the overlap of both upregulated and downregulated IRGs in two independent scores.

### Screening of the Most Significant Modules and IRGs Using WGCNA

**Figure 2:**
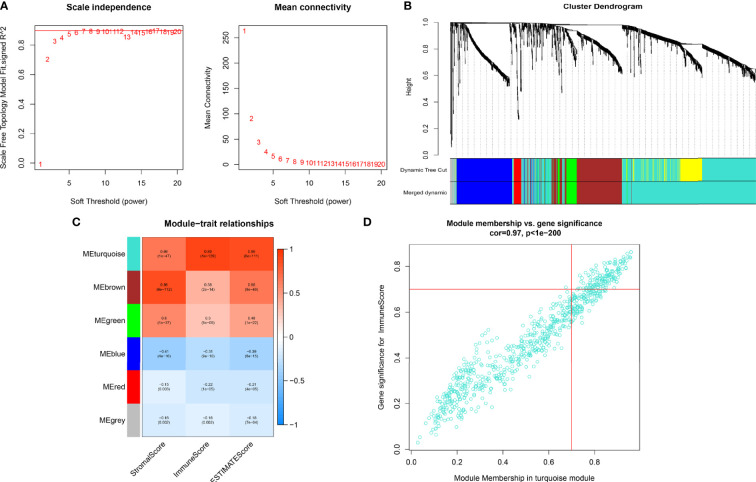
**(A)** In order to achieve a scale-free co-expression network, we chose power index = 4 as the appropriate soft threshold. **(B)** Identification of a gene consensus module. The branches of the dendrogram correspond to four different gene modules. **(C)** Correlation between the gene modules and tumor microenvironment related scores, including immune score, stromal score, and ESTIMATE score. Each cell contains corresponding correlation coefficient and p-value. The correlation coefficient decreased in size from red to blue. **(D)** Scatter plot of module eigengenes in the turquoise module.

WGCNA was utilized to frame a gene co-expression network to identify biologically meaningful gene modules, to further understand the genes causing the differences in OC immune infiltration. A Power index of = 4 was selected as the optimal soft-thresholding parameter after excluding the outlier data (scale-free R^2^ = 0.981) (**Figure 2A**). A scale-free co-expression network was constructed using 1,408 DEGs (**Figure 2B**). Finally, nine modules, CC, and *p* values were obtained (**Figure 2C**). We determined that the turquoise module had the highest correlation with the immune score (CC = 0.89, *p* < 0.001) and ESTIMATE score (CC = 0.86, *p* < 0.001). Therefore, we chose the turquoise module for subsequent analysis.

### Construction of the PRSM Using IRGPs

**Figure 3:**
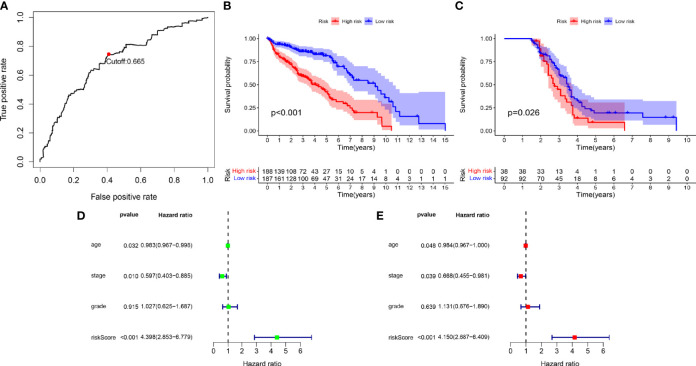
**(A)** Time-dependent receiver operating characteristic (tROC) analysis of the prognostic risk score model. **(B)** Kaplan-Meier curve of overall survival in model group. **(C)** Kaplan-Meier curve of overall survival in validation group. **(D)** Univariate-Cox regression analyze of prognostic factors in model group. **(E)** Multivariate-Cox regression analyze of prognostic factors in model group.

We screened 173 relatively critical IRGs (cor. gene MM > 0.7 and cor. gene GS > 0.7) (**Figure 2D**). The establishment of 14,878 IRGPs was conducted by pairwise alignment of these 173 genes. After the removal of the IRGPs with small variation (0 or 1< 20%), the remaining 771 IRGPs were analyzed using univariate Cox proportional hazards regression. There were significant differences in the 36 IRGPs (*p* < 0.01) (**Supplementary Table S1**). We then performed the analysis of these IRGPs in the model group using Lasso-Cox proportional hazards regression. In the final PRSM, 15 prognostic-related IRGPs and their corresponding risk coefficients were determined (**Table 1**). The RS of each patient in the model group was calculated using the PRSM. We conducted an ROC analysis using the R package “survivalROC” to measure the prognostic ability of the RS model. Based on the 5-year ROC curve, we set the best cut-off value to 0.665 to classify the patients into either the HRG or the LRG (**Figure 3A**). The survival curves of the HRG and LRG indicated that the OS in the HRG was worse than that in the LRG, and the difference between the two groups was statistically significant (*p* < 0.001) (**Figure 3B**). To verify the predictive capability of different datasets, we applied the PRSM to 130 OC samples from GEO (GSE9891) as a validation group. The log-rank test was performed to test the difference in OS between the HRG and LRG compared to the validating groups. The results were consistent with those of the model group, because the OS of the HRG was significantly worse than that of the LRG (*p* = 0.026) (**Figure 3C**). We performed univariate and multivariate Cox regression analyses of RS and clinical variables for OS of OC patients. The results suggested that RS was an independent prognostic factor for OC (**Figures 3D, E**).

**Table 1:** Prognostic risk scoring model information including 15 immune-related gene pairs.

Gene	Coef
BTK|MEF2C	0.29
FERMT3|C5AR1	0.17
SNX20|PIK3R5	0.05
C3AR1|DRAM1	0.07
RCSD1|CXorf21	0.14
PIK3R5|CSF2RB	-0.23
MPEG1|EVI2A	0.40
MPEG1|CD163	0.08
GIMAP6|NPL	0.05
GIMAP6|GLIPR1	0.11
TLR4|LACC1	0.04
DOK3|CSF2RA	-0.18
SLCO2B1|GLIPR1	0.07
GIMAP8|LACC1	0.08
MPP1|GAL3ST4	-0.09

### Immune Infiltration Within Different Risk Groups

**Figure 4:**
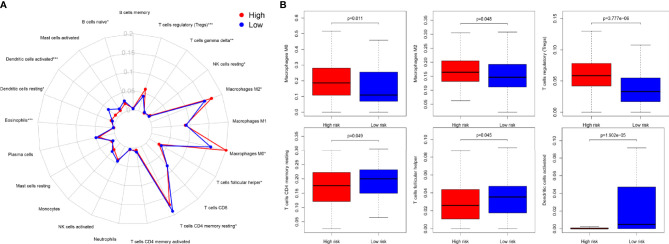
**(A)** Summary of the 22 immune cell types abundance estimated by “CIBERSORT” within different risk groups. **(B)** The differences of 22 immune cell types abundance within different risk groups. Macrophage M0 (*p* = 0.011), macrophage M2 (*p* = 0.048), and Treg cells (*p* < 0.001) were significantly highly expressed in the HSG. Activated memory CD4+ T cells (*p*= 0.049), T follicular helper (*p* = 0.045), and activated dendritic cells (aDC) (*p* < 0.001) were significantly higher in the LSG.

To explore the specific cell types that cause the difference in immune infiltration between the HRG and LRG, we applied “CIBERSORT” to estimate the relative infiltration abundance of immune cell type abundance of 21 types of immune cells in different samples. The Wilcoxon rank- sum test was used to analyze the differences in the contents of various immune cells in the HRG and LRG (**Figure 4A**). The results indicated that macrophage M0 (*p* = 0.011), macrophage M2 (*p* = 0.048), and Tregs (*p* < 0.001) were highly expressed in the HRG. In addition, activated memory CD4^+^ T cells (*p* = 0.049), T follicular helper cells (*p* = 0.045), and activated dendritic cells (aDC) (*p* < 0.001) were highly expressed in the LRG (**Figure 4B**). The results showed specific immune-related reasons for the poor prognosis in the HRG.

### Functional Analysis and Identification of Key IRGPs

**Figure 5:**
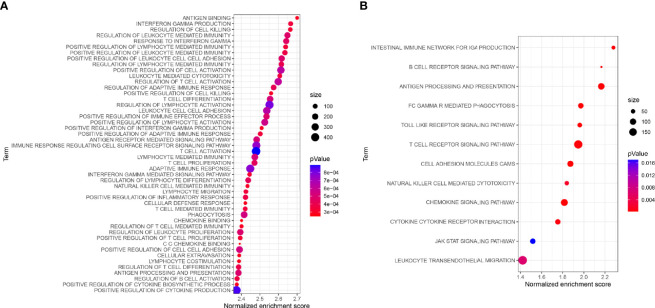
**(A)** GO-related GSEA between different risk groups. **(B)** Functional enrichment analysis of KEGG for key IRGs.

**Figure 6:**
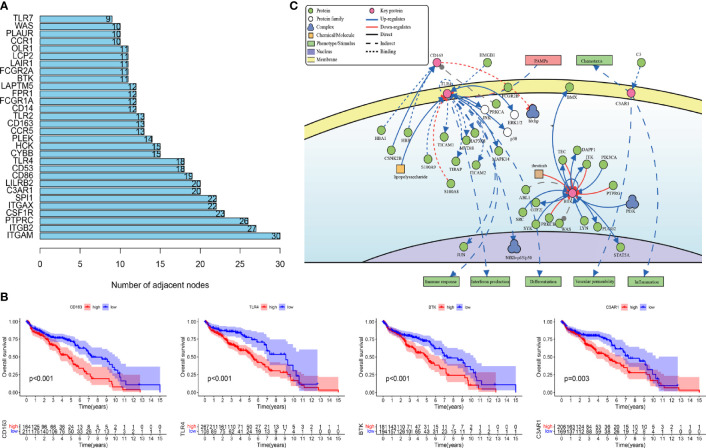
**(A)** Gene nodes ranked among the top 30 in the PPI analyses. PPI, protein-protein interaction. **(B)** Kaplan-Meier curves of overall survival in four prognostic key IRGs. IRGs, immune related genes. **(C)** The causal interaction of key gene analysis in DisNor.

To study the prognosis of the differences between the HRG and LRG in molecular functions, biological processes, and cellular components, we conducted GO-related GSEA (**Figure 5A**). Collectively, these immune-related alterations offer a basis for the molecular mechanism of the PRSM. Through KEGG pathway analysis, we obtained information on the pathways of key IRGPs (**Figure 5B**). We selected the genes with nodes ranked among the top 30 in the selected network that intersected with IRGPs in the PRSM (**Figure 6A**). We then selected genes related to prognosis by plotting Kaplan- Meier survival curves. The key intersection genes included BTK, CD163, TLR4, and C3AR1. In the OS curve, patients in the high expression group had a significantly poorer prognosis than those in the low expression group (all *p* < 0.05) (**Figure 6B**). The DisNor database was used to analyze the upstream and downstream proteins of these four genes and their modes of action. These results strongly suggested that the genes directly interacted with key genes and their binding sites (**Figure 6C**). The results revealed that these key genes were involved in the immune response, inflammation, and vascular penetration.

### RT-qPCR Analysis of the Candidate Genes

**Figure 7:**
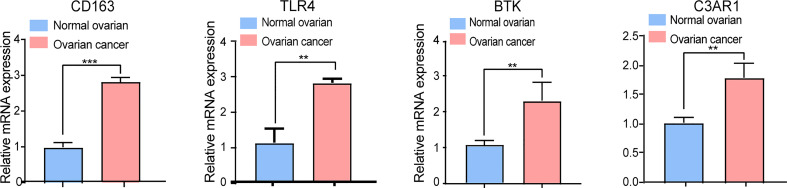
RT-qPCR analysis of four key IRGs in the ovarian cancer tissues and normal ovarian tissues. All experiments were performed in triplicate. **p- value t-test < 0.01; ***p- value t-test < 0.001.

To explore whether the expression of these candidate genes was altered in OC, we performed RT-qPCR on OC tissues and normal ovarian tissues. As shown in **Figure 7**, all four key prognostic IRGs displayed meaningful results in the RT-qPCR assay. These four key prognostic IRGs were expressed at low levels in normal tissues and were highly expressed in tumor tissues (*p* < 0.01). This result is in line with our previous results from TCGA and GEO validation.

### Sensitivity Analyses of Different Treatments

**Figure 8:**
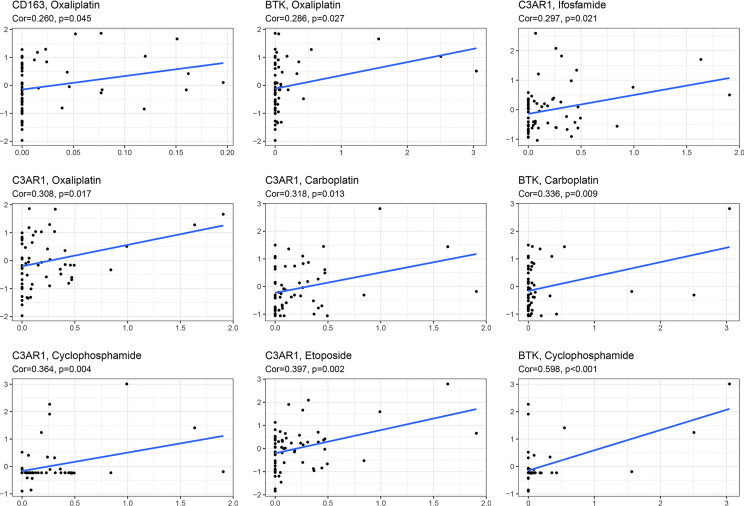
Sensitivity analysis of key IRGs expression within different chemotherapeutic drugs.

Chemotherapy is one of the most important therapeutic methods for treating OC. Gene expression data and response data for chemotherapy drugs were downloaded from CellMiner™ (https://discover.nci.nih.gov/cellminer/). The results revealed a positive correlation between the CD163 expression pattern and oxaliplatin efficacy (*p* < 0.05). The higher the expression of BTK, the better the therapeutic effect of oxaliplatin, carboplatin, and cyclophosphamide (*p* < 0.05). The expression of C3AR1 was more positively correlated with the therapeutic effects of oxaliplatin, carboplatin, etoposide, ifosfamide, and cyclophosphamide (*p* < 0.05) (**Figure 8**).

**Figure 9:**
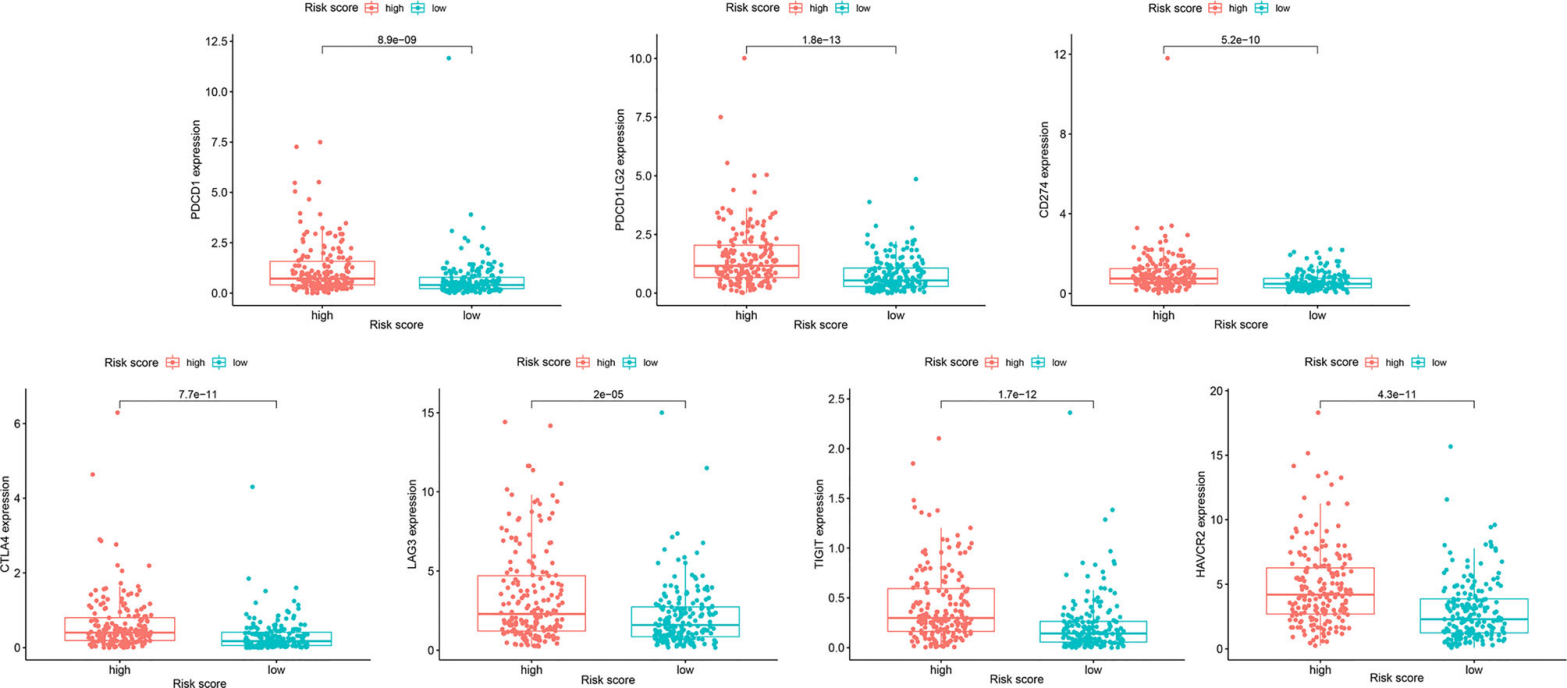
The expression differences of 7 immunosuppressive checkpoint genes in HRG and LRG.

**Figure 10:**
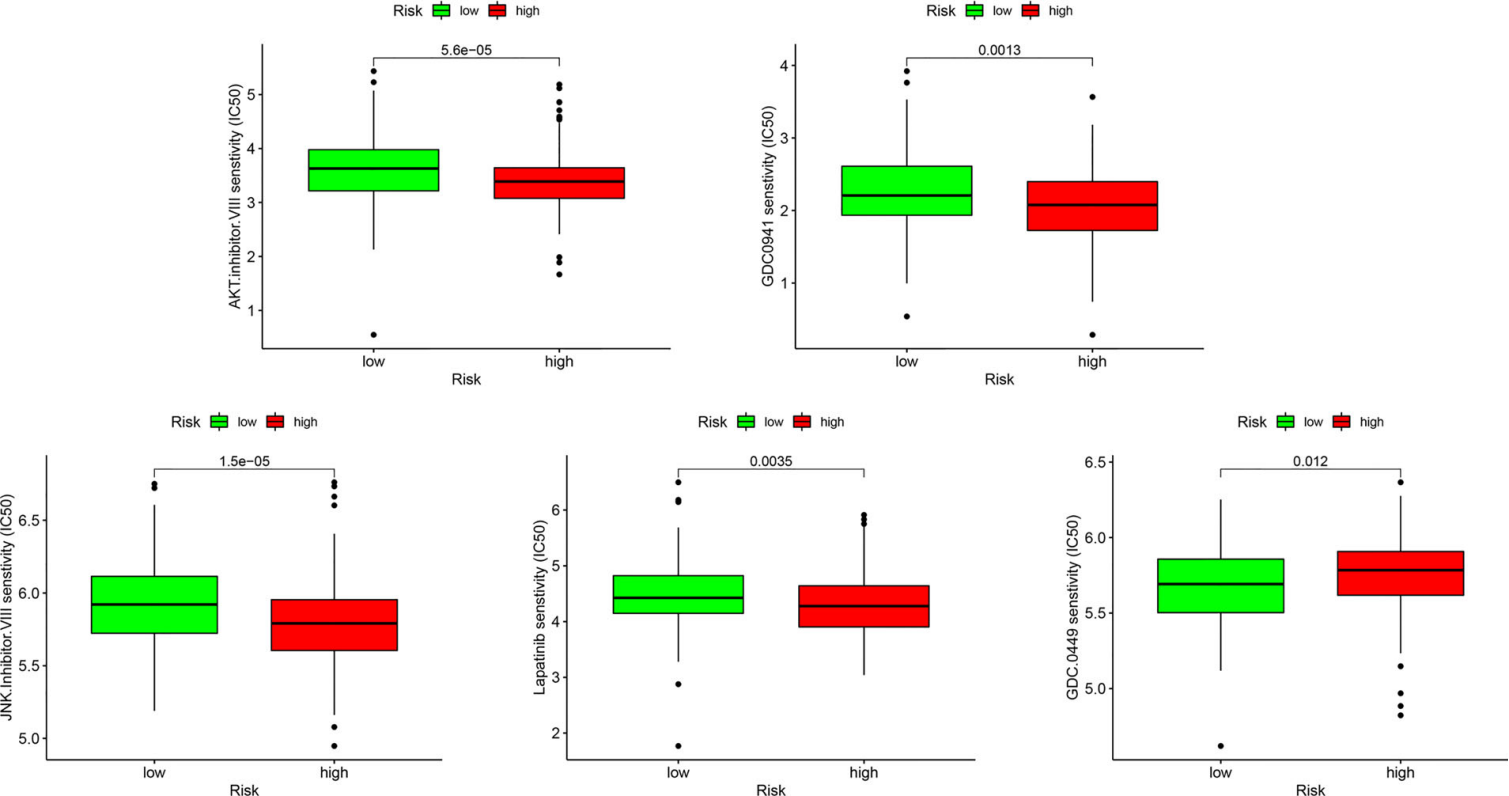
The sensitivity difference of multiple targeted inhibitors within different risk groups, including AKT inhibitor VIII, GDC0941, JNK Inhibitor VIII, Lapatinib, and GDC-0449.

Because resistance to chemotherapy drugs limits their therapeutic effect, immunotherapy is an emerging treatment method for OC. Recently, immune checkpoint inhibitors (ICIs) have been identified as promising cancer immunotherapeutic approaches. Therefore, based on the immunophenotypic score, we evaluated seven important immunosuppressive checkpoints. We verified the expression of these immune suppression checkpoints in the HRG and LRG, confirmed that they were all highly expressed in the HRG (**Figure 9**). These seven immune genes are expected to become immune suppression checkpoints. In addition, we separately calculated the IC50 concentrations of AKT inhibitor VIII, GDC0941, JNK inhibitor VIII, lapatinib, and GDC-0449 in the HRG and LRG. Except for GDC0449, other inhibitors had lower IC50 concentrations in the HRG (*p* < 0.05) (**Figure 10**). These results showed that the key IRGs we screened may be potential immunotherapy targets. When their expression is high, the effect of treatment is better, which provides a basis for the targeted therapy in patients.

**Figure 11:**
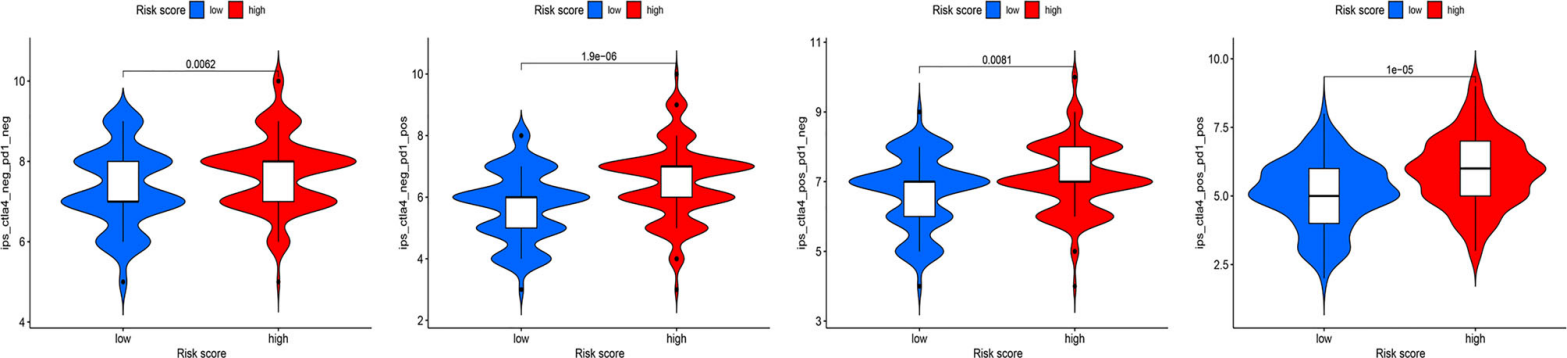
The association between IPS and risk groups of OC patients.

Recent studies have shown that IPS can predict the therapeutic effects of ICIs in cancer patients. This was based on the existing high immunogenic potential. We applied the immunophenotypic score to compare the HRG and LRG after applying different ICIs (**Figure 11**). As shown in the figure, regardless of whether cytotoxic T-lymphocyte antigen 4 (CTLA-4) or programmed cell death protein 1 (PD-1) was used for treatment, the immunophenotypic score of the HRG was higher than that of the LRG. This finding indicated that treatment with ICIs was more effective for patients in the HRG.

## Discussion

OC is the second most common gynecological malignancy, with a high recurrence rate and chemoresistance. Primary debulking surgery followed by chemotherapy is the standard treatment for OC (19, 20). The application of anti-angiogenic drugs and targeted drugs has been applied in recent years (21, 22). However, these treatment options are still not ideal for improving patient survival. In recent years, immune cell-based treatment has become a promising method that can better treat and potentially cure malignant tumors that are difficult to cure using chemotherapy, surgery, or radiotherapy (23–25). Immunotherapy of OC has made considerable progress in the past two decades, such as with the use of PD-1 targeted therapy (26). However, some patients cannot benefit from immunotherapy, which may be due to the tumor immunosuppressive microenvironment (27, 28). Therefore, searching for differentially expressed immune-related biomarkers in the tumor immune microenvironment can provide important prognostic value and regulatory targets for immunotherapy, and provide a molecular basis for immunotherapy.

In the present study, based on the OC immune-related gene dataset, we screened IRGPs related to the OS of patients through the use of a PRSM. To avoid deviation of the results caused by a single database, we used the GEO database for verification. By analyzing the differences in immune cell infiltration between the HRG and LRG, we found that Tregs, M0 macrophages, and M2 macrophages had significantly high infiltration in the HRG. This infiltration of immune cells might favorably change the immunosuppressive status of the tumor microenvironment, as well as pathways involved in tumor metastasis and invasion (29, 30). It has been demonstrated that immune suppressor cells, including Tregs, M0 macrophages, and M2 macrophages, are associated with poorer outcomes (31, 32). Tregs induce T cell cycle arrest (33), produce granzyme and perforin to kill T cells (34), release cytokines, inhibit the expression of antigen-presenting cells, CD80, and CD86 (35), and directly inhibit T cell activation and promote tumorigenesis. In addition, a number of studies have demonstrated that M2 macrophages had tumor-promoting properties (36) and Stat6 was the major transcription factor responsible for the induction of M2 genes (37). Associated with this, we observed strong enrichment for STAT pathway members in GSEA. In a recent study, Izar et al. demonstrated that JAK STAT pathway activation and the enrichment of M2 macrophages in high-grade serous OC were associated with poor prognosis through single-cell sequencing technology (38). In the LRG, T follicular helper cells, activated memory CD4^+^ T cells, and dendritic cells were highly expressed. Tfh cells are the key to enhancing the immune response and understanding their functions will help in the development of vaccines. In addition, in many studies, CD4^+^ T cells have been shown to enhance antitumor immune function by regulating dendritic cells or stimulating other pro-inflammatory myeloid cells (39–41). These findings provide strong evidence that tumor-infiltrating immune cells have prognostic value in patients.

In our study, we screened four prognostic key IRGs from the PRSM, in which CD163 and TLR4 are type I transmembrane proteins, BTK is a key regulator of the B-cell receptor signaling pathway, and C3AR1 is a transmembrane G protein-coupled receptor protein with seven membrane-spanning domains. CD163 is considered to be a highly specific marker of M2 macrophages, which is a scavenger receptor expressed on monocytes and macrophages (42). CD163 can be used as an immune modulator and aids in the inflammatory response (43), and as a member of the tumor-associated macrophage family, it has an important impact on tumor proliferation and metastasis (44). TLR4 triggers immune responses *via* the TLR4 signaling pathway (45, 46) and promotes tumor development and progression *via* pro-inflammatory responses (47). BTK is a member of the Tec family of tyrosine kinases. As a component of the TLR pathway, BTK plays an important role in innate and adaptive immune functions (48). BTK inhibitors are currently approved by the FDA for the treatment of lymphoma and leukemia (49). Complement C3a is important in the regulation of immune response as well as in organ inflammation and injury (50). C3a/C3aR (C3a receptor) signaling promotes tumor growth by promoting immunosuppression through modulated tumor-associated macrophages, thereby repressing antitumor immunity (51). C3aR1 has been shown to be expressed abnormally in a variety of human cancers, and it predicts resistance to chemoradiation and poor prognosis in osteosarcoma (52). Although these IRGs are not exactly the same, they were all mainly enriched in pathways closely associated with the microenvironment and could predict the prognosis of related cancers. Therefore, the above studies have shown that these genes are key genes that affect prognosis, and should hopefully become targets for immunotherapy.

A series of studies have shown that ICIs are of great significance in the treatment of OC (4). However, only a small percentage of cancer patients respond to immunotherapy, presumably because of differences in the immunophenotypic, and tumor microenvironment characteristics (53). Thus, it is important to identify biomarkers for immune checkpoint blockade therapies in this specific setting. With the advent of the era of precision medicine, the model for assessing disease with a single prognostic marker has gradually been abandoned. Therefore, efforts to develop an effective immune-related model to provide a more adequate basis for evaluating the therapeutic effect of patients have been increased. We analyzed the relationship between IRG expression and chemotherapy sensitivity and found that the expression of the four IRGs we screened was positively correlated with chemotherapy sensitivity. Another important result was that the expression levels of immune checkpoints in the high-risk cohort were significantly higher than those in the low-risk cohort, which was consistent with the positive correlation between the RS and the expression level of immune checkpoints. Additionally, in the determination of the IC50 of the TIs, we found that the HRG was more sensitive to these drugs. However, owning to no published data regarding immunotherapy in ovarian cancer, we need other methods to predict the sensitivity of immunotherapies. Zlatko et al. created The Cancer Immunome Atlas (https://tcia.at/) and developed a scoring scheme for the quantification termed IPS (54). In this publication, IPS as a scoring scheme for solid cancers was a predictor of response to checkpoint blockade. We downloaded the IPS of patients with OC from the TCIA database (https://tcia.at/). The Cancer Immunome Atlas (TCIA) can be queried for the gene expression of specific immune-related gene sets, cellular composition of immune infiltrates, neoantigens and cancer-germline antigens, HLA types, and tumor heterogeneity. The IPS was proved to be a predictor of response to checkpoint blockers in patients with melanoma. IPS may serve as a useful tool for evaluating the efficacy of ICIs (55) and this view was verified in recent study. Furthermore, the close associations of sensitivity to tumor immunotherapy with immune checkpoint genes and tumor immune infiltration (56, 57). By compared the IPS between the HRG and LRG in different immunotherapy decisions, we found the immunophenotypic score of the HRG was higher than that of the LRG. We infer that these signatures were able to predict the sensitivity of immunotherapies *via* the methods described above.

Despite these promising results, this study has some limitations. First, the PRSM, established based on gene expression, was formed based on limited data from retrospective studies. Thus, a large number of studies are required to explore the specific functions of these key prognostic IRGs. Second, although our results uncovered these four IRGs as potentially useful biomarkers, these data will need to be further validated in large, prospective clinical trials.

In summary, we constructed a PRSM based on the difference in IRGs between the HRG and LRG. Among the differentially expressed IRGs, four key genes were identified through analysis of their prognostic impact. Through the study of these genes, we have a deeper understanding of immune-related mechanisms. These genes may be potential predictive markers for immunotherapy and immunotherapeutic targets, which in turn may open up a new chapter in OC immunotherapy.

## Data Availability Statement

The datasets presented in this study can be found in online repositories. The names of the repository/repositories and accession number(s) can be found in the article/**Supplementary Material**.

## Ethics Statement

The studies involving human participants were reviewed and approved by Ethics Committee of Affiliated Tumor Hospital of Harbin Medical University. The patients/participants provided their written informed consent to participate in this study. Written informed consent was obtained from the individual(s) for the publication of any potentially identifiable images or data included in this article.

## Author Contributions

B-BC, CY, and GL designed the manuscript. Z-CZ, J-NG and NZ drafted the manuscript. Z-CZ and J-NG drew figures and tables. Z-QW, CY, and B-BC revised the manuscript. GL made several revisions to the manuscript. All authors contributed to the article and approved the submitted version.

## Funding

This work was supported by the National Natural Science Foundation of China (nos. 81872507), Key Program of Natural Science Foundation of Heilongjiang Province (ZD2020H007), the Nn10 program (Nn10py2017-01), and the fund of the Cancer Hospital, Harbin Medical University, China (JJZD2017-01).

## Conflict of Interest

The authors declare that the research was conducted in the absence of any commercial or financial relationships that could be construed as a potential conflict of interest.

## Publisher’s Note

All claims expressed in this article are solely those of the authors and do not necessarily represent those of their affiliated organizations, or those of the publisher, the editors and the reviewers. Any product that may be evaluated in this article, or claim that may be made by its manufacturer, is not guaranteed or endorsed by the publisher.

## Abbreviations

OC, ovarian cancer; ICIs, immune checkpoint inhibitors; PRSM, prognostic risk scoring model; TCGA, The Cancer Genome Atlas; GEO, Gene Expression Omnibus; WGCNA, weighted gene co-expression network analysis; IRGs, immune-related genes; TIs, targeted inhibitors; HRG, high-risk group; LRG, low-risk group; RS, risk score; DEGs, differentially expressed genes; AM, adjacency matrix; TOM, topological overlap matrix; CC, correlation coefficient; GS, gene significance; TLR4, Toll-like receptor 4; BTK, Bruton’s tyrosine kinase; CTLA-4, cytotoxic T-lymphocyte antigen 4; PD-1, programmed cell death protein 1; MM, module membership; ROC, receiver operating characteristics; GO, Gene Ontology; KEGG, Kyoto Encyclopedia of Genes and Genomes; GSEA, gene set enrichment analysis; HH, Hedgehog; OS, overall survival; aDC, activated dendritic cells.
