# Supplementary material for: Identification and Validation of Immune-Related Gene for Predicting Prognosis and Therapeutic Response in Ovarian Cancer
Source: Front Immunol. 2021 Nov 22;12:763791. doi: 10.3389/fimmu.2021.763791 (PMC8645858; doi:10.3389/fimmu.2021.763791)
Supplement: Supplementary file 1 [file Table_1.docx]

Table.S1. A total of 36 IRGPs were selected by univariate-Cox proportional hazards regression.

| gene | HR | HR.95L | HR.95H | Cox *P* value |
| --- | --- | --- | --- | --- |
| MPEG1\|EVI2A | 2.30 | 1.50 | 3.53 | ＜0.001 |
| BTK\|MEF2C | 2.01 | 1.40 | 2.90 | ＜0.001 |
| SLCO2B1\|GLIPR1 | 2.09 | 1.39 | 3.15 | ＜0.001 |
| GIMAP6\|GLIPR1 | 1.78 | 1.23 | 2.57 | 0.002 |
| TLR4\|LACC1 | 1.71 | 1.21 | 2.41 | 0.002 |
| GIMAP8\|LACC1 | 1.66 | 1.20 | 2.32 | 0.003 |
| FERMT3\|C5AR1 | 1.69 | 1.18 | 2.44 | 0.005 |
| TAGAP\|LACC1 | 1.61 | 1.16 | 2.25 | 0.005 |
| PLEK\|C5AR1 | 1.58 | 1.14 | 2.20 | 0.006 |
| GIMAP6\|NPL | 1.65 | 1.15 | 2.37 | 0.007 |
| PIK3R5\|CSF2RB | 0.56 | 0.37 | 0.86 | 0.008 |
| C3AR1\|DRAM1 | 1.68 | 1.13 | 2.48 | 0.010 |
| MPEG1\|PECAM1 | 1.61 | 1.12 | 2.31 | 0.010 |
| GIMAP6\|ELL2 | 1.68 | 1.12 | 2.53 | 0.013 |
| CD84\|PTPRE | 1.50 | 1.08 | 2.08 | 0.016 |
| PLEK\|LY86 | 1.46 | 1.05 | 2.04 | 0.024 |
| MPEG1\|CD163 | 1.47 | 1.05 | 2.04 | 0.024 |
| FPR1\|PTPRE | 1.51 | 1.05 | 2.19 | 0.028 |
| RCSD1\|PTPRE | 1.46 | 1.04 | 2.05 | 0.028 |
| CLEC4A\|ACVRL1 | 1.46 | 1.04 | 2.05 | 0.028 |
| PLEK\|LY96 | 1.48 | 1.04 | 2.10 | 0.031 |
| PLEK\|DRAM1 | 1.45 | 1.02 | 2.05 | 0.038 |
| HCK\|MPEG1 | 0.69 | 0.48 | 0.98 | 0.040 |
| DOK3\|CSF2RA | 0.68 | 0.47 | 0.99 | 0.042 |
| CSF2RA\|PTPRE | 1.40 | 1.00 | 1.97 | 0.047 |
| FERMT3\|MPEG1 | 0.69 | 0.47 | 1.00 | 0.053 |
| SNX20\|PIK3R5 | 1.39 | 0.99 | 1.93 | 0.054 |
| RCSD1\|CXorf21 | 1.45 | 0.99 | 2.12 | 0.056 |
| EVI2B\|GIMAP4 | 1.43 | 0.99 | 2.06 | 0.057 |
| SLCO2B1\|STK10 | 1.39 | 0.99 | 1.95 | 0.060 |
| HCK\|C5AR1 | 1.39 | 0.98 | 1.96 | 0.061 |
| PTPRC\|PTPRE | 1.48 | 0.98 | 2.25 | 0.062 |
| MPP1\|GAL3ST4 | 0.66 | 0.42 | 1.03 | 0.068 |
| MPEG1\|CCR1 | 1.38 | 0.98 | 1.95 | 0.069 |
| TLR4\|APOBR | 1.35 | 0.97 | 1.88 | 0.076 |
| STX11\|LACC1 | 1.37 | 0.96 | 1.97 | 0.084 |
