# Supplementary material for: Identification and Validation of Immune-Related Gene for Predicting Prognosis and Therapeutic Response in Ovarian Cancer
Source: Front Immunol. 2021 Nov 22;12:763791. doi: 10.3389/fimmu.2021.763791 (PMC8645858; doi:10.3389/fimmu.2021.763791)
Supplement: Supplementary file 2 [file Table_2.docx]

Table.S2. Risk grouping of patients in model and validation groups.

| ID | Cohort | Risk Score | Risk Groups |
| --- | --- | --- | --- |
| TCGA-OY-A56Q | TCGA | 0.999 | high |
| TCGA-24-1567 | TCGA | 0.223 | low |
| TCGA-10-0938 | TCGA | 0.019 | low |
| TCGA-13-1488 | TCGA | -0.014 | low |
| TCGA-09-2051 | TCGA | 1.197 | high |
| TCGA-30-1714 | TCGA | 0.211 | low |
| TCGA-13-0730 | TCGA | 0.485 | low |
| TCGA-29-1769 | TCGA | 0.278 | low |
| TCGA-13-0900 | TCGA | 0.033 | low |
| TCGA-36-1581 | TCGA | 1.377 | high |
| TCGA-23-1118 | TCGA | 0.491 | low |
| TCGA-61-2003 | TCGA | 1.036 | high |
| TCGA-04-1542 | TCGA | 0.095 | low |
| TCGA-24-1431 | TCGA | 0.702 | high |
| TCGA-13-0885 | TCGA | 0.998 | high |
| TCGA-04-1651 | TCGA | 0.459 | low |
| TCGA-29-1696 | TCGA | 0.604 | low |
| TCGA-25-1626 | TCGA | 0.444 | low |
| TCGA-24-0982 | TCGA | 0.121 | low |
| TCGA-61-2098 | TCGA | 0.600 | low |
| TCGA-09-0364 | TCGA | 0.798 | high |
| TCGA-13-0920 | TCGA | 0.307 | low |
| TCGA-13-0891 | TCGA | 0.292 | low |
| TCGA-13-1509 | TCGA | -0.111 | low |
| TCGA-24-1557 | TCGA | 0.757 | high |
| TCGA-13-1512 | TCGA | 0.829 | high |
| TCGA-13-1497 | TCGA | 0.474 | low |
| TCGA-13-0905 | TCGA | 0.124 | low |
| TCGA-23-2078 | TCGA | 1.008 | high |
| TCGA-25-2400 | TCGA | 0.499 | low |
| TCGA-23-1119 | TCGA | 0.278 | low |
| TCGA-13-2060 | TCGA | 0.554 | low |
| TCGA-13-1511 | TCGA | 0.344 | low |
| TCGA-24-1464 | TCGA | 0.021 | low |
| TCGA-31-1959 | TCGA | 1.008 | high |
| TCGA-29-1768 | TCGA | 0.256 | low |
| TCGA-25-2396 | TCGA | 1.007 | high |
| TCGA-25-1315 | TCGA | 0.610 | low |
| TCGA-24-1850 | TCGA | 1.189 | high |
| TCGA-13-1489 | TCGA | 0.012 | low |
| TCGA-24-1427 | TCGA | 0.850 | high |
| TCGA-23-1122 | TCGA | 0.472 | low |
| TCGA-25-1623 | TCGA | 0.906 | high |
| TCGA-29-1693 | TCGA | 0.404 | low |
| TCGA-09-2045 | TCGA | 0.602 | low |
| TCGA-24-1550 | TCGA | 0.619 | low |
| TCGA-57-1994 | TCGA | 1.034 | high |
| TCGA-24-2035 | TCGA | 0.964 | high |
| TCGA-24-1549 | TCGA | 1.112 | high |
| TCGA-23-1111 | TCGA | 0.766 | high |
| TCGA-24-1425 | TCGA | 0.785 | high |
| TCGA-VG-A8LO | TCGA | 0.167 | low |
| TCGA-31-1951 | TCGA | 0.956 | high |
| TCGA-25-1317 | TCGA | 0.420 | low |
| TCGA-57-1582 | TCGA | 0.703 | high |
| TCGA-25-1320 | TCGA | 0.474 | low |
| TCGA-13-1495 | TCGA | 0.133 | low |
| TCGA-24-1470 | TCGA | 1.556 | high |
| TCGA-61-1724 | TCGA | 0.469 | low |
| TCGA-59-2354 | TCGA | 1.046 | high |
| TCGA-24-1560 | TCGA | 0.396 | low |
| TCGA-20-1682 | TCGA | 0.437 | low |
| TCGA-04-1514 | TCGA | 0.209 | low |
| TCGA-29-1762 | TCGA | 0.639 | low |
| TCGA-36-1568 | TCGA | 1.268 | high |
| TCGA-10-0931 | TCGA | 0.222 | low |
| TCGA-24-1842 | TCGA | 1.162 | high |
| TCGA-25-2393 | TCGA | 0.790 | high |
| TCGA-13-0887 | TCGA | -0.003 | low |
| TCGA-29-1781 | TCGA | 1.377 | high |
| TCGA-59-2363 | TCGA | 0.731 | high |
| TCGA-36-1569 | TCGA | 1.213 | high |
| TCGA-09-0366 | TCGA | 0.783 | high |
| TCGA-29-1694 | TCGA | 0.746 | high |
| TCGA-13-0804 | TCGA | 0.264 | low |
| TCGA-61-1741 | TCGA | 1.034 | high |
| TCGA-29-1761 | TCGA | 0.649 | low |
| TCGA-23-1027 | TCGA | 0.397 | low |
| TCGA-57-1993 | TCGA | 0.831 | high |
| TCGA-04-1343 | TCGA | 0.159 | low |
| TCGA-10-0927 | TCGA | 0.536 | low |
| TCGA-61-1995 | TCGA | 1.450 | high |
| TCGA-24-0968 | TCGA | 0.437 | low |
| TCGA-24-1565 | TCGA | 0.626 | low |
| TCGA-24-2254 | TCGA | 0.618 | low |
| TCGA-61-2092 | TCGA | 0.706 | high |
| TCGA-23-1109 | TCGA | 1.151 | high |
| TCGA-24-1417 | TCGA | 1.330 | high |
| TCGA-13-1501 | TCGA | 0.025 | low |
| TCGA-10-0928 | TCGA | 0.786 | high |
| TCGA-24-1552 | TCGA | 0.349 | low |
| TCGA-13-0720 | TCGA | -0.071 | low |
| TCGA-24-1616 | TCGA | 0.494 | low |
| TCGA-25-1319 | TCGA | 1.034 | high |
| TCGA-24-1603 | TCGA | 1.041 | high |
| TCGA-13-1485 | TCGA | 0.081 | low |
| TCGA-30-1866 | TCGA | 0.518 | low |
| TCGA-61-1914 | TCGA | 0.155 | low |
| TCGA-61-2113 | TCGA | 0.641 | low |
| TCGA-36-1574 | TCGA | 1.393 | high |
| TCGA-24-1418 | TCGA | 0.254 | low |
| TCGA-24-1930 | TCGA | 1.322 | high |
| TCGA-24-1845 | TCGA | 0.405 | low |
| TCGA-24-2293 | TCGA | 0.850 | high |
| TCGA-13-0888 | TCGA | -0.136 | low |
| TCGA-25-1322 | TCGA | 0.641 | low |
| TCGA-20-0991 | TCGA | 1.204 | high |
| TCGA-24-1105 | TCGA | 0.469 | low |
| TCGA-29-1776 | TCGA | 1.153 | high |
| TCGA-24-1562 | TCGA | 0.698 | high |
| TCGA-23-1028 | TCGA | 0.872 | high |
| TCGA-57-1585 | TCGA | 0.678 | high |
| TCGA-24-1471 | TCGA | 0.695 | high |
| TCGA-24-1422 | TCGA | 0.796 | high |
| TCGA-23-1029 | TCGA | 0.794 | high |
| TCGA-13-1505 | TCGA | 0.451 | low |
| TCGA-24-1928 | TCGA | 1.097 | high |
| TCGA-04-1338 | TCGA | -0.056 | low |
| TCGA-04-1356 | TCGA | 0.118 | low |
| TCGA-13-0908 | TCGA | 0.846 | high |
| TCGA-25-1321 | TCGA | 0.757 | high |
| TCGA-13-0911 | TCGA | 0.753 | high |
| TCGA-13-0725 | TCGA | -0.135 | low |
| TCGA-61-1736 | TCGA | 0.662 | low |
| TCGA-24-1604 | TCGA | 0.863 | high |
| TCGA-24-2027 | TCGA | 0.507 | low |
| TCGA-13-0766 | TCGA | -0.124 | low |
| TCGA-23-1107 | TCGA | 0.406 | low |
| TCGA-24-1419 | TCGA | 0.564 | low |
| TCGA-24-1551 | TCGA | 0.916 | high |
| TCGA-13-0897 | TCGA | 0.182 | low |
| TCGA-23-1030 | TCGA | 0.360 | low |
| TCGA-13-A5FT | TCGA | 0.521 | low |
| TCGA-31-1950 | TCGA | 1.008 | high |
| TCGA-29-1785 | TCGA | 1.474 | high |
| TCGA-23-1110 | TCGA | 0.885 | high |
| TCGA-13-1492 | TCGA | -0.269 | low |
| TCGA-25-2409 | TCGA | 1.213 | high |
| TCGA-25-1318 | TCGA | 0.393 | low |
| TCGA-09-1666 | TCGA | 1.007 | high |
| TCGA-04-1364 | TCGA | 0.239 | low |
| TCGA-09-2048 | TCGA | 0.067 | low |
| TCGA-61-2088 | TCGA | 0.406 | low |
| TCGA-24-2038 | TCGA | 0.746 | high |
| TCGA-25-1633 | TCGA | 0.931 | high |
| TCGA-09-1670 | TCGA | 0.979 | high |
| TCGA-20-1687 | TCGA | 1.268 | high |
| TCGA-25-1329 | TCGA | 0.641 | low |
| TCGA-30-1853 | TCGA | 0.065 | low |
| TCGA-23-1021 | TCGA | 0.229 | low |
| TCGA-24-1435 | TCGA | 0.693 | high |
| TCGA-04-1332 | TCGA | 0.953 | high |
| TCGA-04-1655 | TCGA | -0.188 | low |
| TCGA-24-1428 | TCGA | 0.727 | high |
| TCGA-13-1498 | TCGA | 1.114 | high |
| TCGA-25-1312 | TCGA | 0.718 | high |
| TCGA-04-1648 | TCGA | 0.427 | low |
| TCGA-13-1403 | TCGA | -0.129 | low |
| TCGA-13-1507 | TCGA | 0.665 | low |
| TCGA-23-2077 | TCGA | 1.089 | high |
| TCGA-13-1510 | TCGA | 0.131 | low |
| TCGA-25-2401 | TCGA | 1.275 | high |
| TCGA-13-0727 | TCGA | 0.032 | low |
| TCGA-04-1536 | TCGA | -0.190 | low |
| TCGA-29-1774 | TCGA | 0.559 | low |
| TCGA-24-1430 | TCGA | 0.768 | high |
| TCGA-61-2097 | TCGA | 0.703 | high |
| TCGA-29-2414 | TCGA | 0.135 | low |
| TCGA-13-1499 | TCGA | 0.585 | low |
| TCGA-20-0987 | TCGA | 1.026 | high |
| TCGA-24-2020 | TCGA | 0.650 | low |
| TCGA-29-1703 | TCGA | 0.553 | low |
| TCGA-25-1634 | TCGA | 0.080 | low |
| TCGA-24-2271 | TCGA | 1.295 | high |
| TCGA-5X-AA5U | TCGA | 0.723 | high |
| TCGA-24-1558 | TCGA | 0.959 | high |
| TCGA-23-1022 | TCGA | 0.524 | low |
| TCGA-61-1998 | TCGA | 0.798 | high |
| TCGA-13-1487 | TCGA | -0.243 | low |
| TCGA-10-0937 | TCGA | 0.432 | low |
| TCGA-36-1576 | TCGA | 1.241 | high |
| TCGA-61-2104 | TCGA | 1.089 | high |
| TCGA-61-1733 | TCGA | 0.033 | low |
| TCGA-36-1580 | TCGA | 0.964 | high |
| TCGA-23-2084 | TCGA | 0.492 | low |
| TCGA-25-1328 | TCGA | 0.800 | high |
| TCGA-24-1847 | TCGA | 1.007 | high |
| TCGA-13-0901 | TCGA | 0.120 | low |
| TCGA-04-1331 | TCGA | -0.128 | low |
| TCGA-13-1404 | TCGA | 0.545 | low |
| TCGA-23-1120 | TCGA | 0.863 | high |
| TCGA-59-A5PD | TCGA | 0.537 | low |
| TCGA-13-0886 | TCGA | 0.340 | low |
| TCGA-24-2033 | TCGA | -0.280 | low |
| TCGA-29-1695 | TCGA | 0.903 | high |
| TCGA-09-0367 | TCGA | 1.439 | high |
| TCGA-13-0797 | TCGA | 1.162 | high |
| TCGA-24-1423 | TCGA | 1.151 | high |
| TCGA-09-1661 | TCGA | 0.020 | low |
| TCGA-29-1778 | TCGA | 1.034 | high |
| TCGA-23-1026 | TCGA | 1.012 | high |
| TCGA-13-1408 | TCGA | 0.184 | low |
| TCGA-59-2350 | TCGA | 0.866 | high |
| TCGA-61-2111 | TCGA | 0.718 | high |
| TCGA-24-1474 | TCGA | 0.564 | low |
| TCGA-31-1946 | TCGA | 1.021 | high |
| TCGA-13-1411 | TCGA | 1.295 | high |
| TCGA-24-1416 | TCGA | 0.695 | high |
| TCGA-24-1424 | TCGA | 0.734 | high |
| TCGA-61-2109 | TCGA | 0.457 | low |
| TCGA-13-0768 | TCGA | 0.484 | low |
| TCGA-59-2348 | TCGA | 0.381 | low |
| TCGA-29-1711 | TCGA | 1.295 | high |
| TCGA-24-1553 | TCGA | 1.268 | high |
| TCGA-13-1409 | TCGA | -0.083 | low |
| TCGA-13-0800 | TCGA | 0.301 | low |
| TCGA-29-A5NZ | TCGA | 0.485 | low |
| TCGA-29-1690 | TCGA | 0.510 | low |
| TCGA-09-2044 | TCGA | 1.096 | high |
| TCGA-25-1316 | TCGA | 1.046 | high |
| TCGA-24-2280 | TCGA | 0.635 | low |
| TCGA-24-1467 | TCGA | 0.750 | high |
| TCGA-24-1563 | TCGA | 0.169 | low |
| TCGA-13-0906 | TCGA | 0.044 | low |
| TCGA-13-0913 | TCGA | 0.524 | low |
| TCGA-61-1725 | TCGA | 0.551 | low |
| TCGA-25-1323 | TCGA | 0.268 | low |
| TCGA-59-2355 | TCGA | 0.637 | low |
| TCGA-61-1911 | TCGA | 1.260 | high |
| TCGA-36-1571 | TCGA | 0.482 | low |
| TCGA-61-1738 | TCGA | 0.369 | low |
| TCGA-09-1662 | TCGA | 1.295 | high |
| TCGA-30-1861 | TCGA | 0.381 | low |
| TCGA-61-2101 | TCGA | 0.668 | high |
| TCGA-04-1362 | TCGA | 0.447 | low |
| TCGA-13-1496 | TCGA | 0.176 | low |
| TCGA-29-2428 | TCGA | 1.377 | high |
| TCGA-24-1413 | TCGA | 0.407 | low |
| TCGA-24-1924 | TCGA | 1.010 | high |
| TCGA-13-0762 | TCGA | -0.112 | low |
| TCGA-09-1668 | TCGA | 0.778 | high |
| TCGA-29-1688 | TCGA | 0.745 | high |
| TCGA-24-2036 | TCGA | 0.557 | low |
| TCGA-13-0883 | TCGA | 0.504 | low |
| TCGA-24-2298 | TCGA | 0.017 | low |
| TCGA-25-1631 | TCGA | 0.275 | low |
| TCGA-04-1347 | TCGA | -0.269 | low |
| TCGA-23-1023 | TCGA | 0.953 | high |
| TCGA-24-1426 | TCGA | 0.349 | low |
| TCGA-13-0924 | TCGA | 1.260 | high |
| TCGA-61-1919 | TCGA | 0.984 | high |
| TCGA-13-0916 | TCGA | 1.172 | high |
| TCGA-24-1923 | TCGA | -0.114 | low |
| TCGA-31-1944 | TCGA | 0.636 | low |
| TCGA-61-1918 | TCGA | 0.979 | high |
| TCGA-25-2404 | TCGA | 0.850 | high |
| TCGA-09-1669 | TCGA | 0.998 | high |
| TCGA-25-1632 | TCGA | 0.659 | low |
| TCGA-25-1326 | TCGA | 0.703 | high |
| TCGA-09-1659 | TCGA | 0.807 | high |
| TCGA-29-1701 | TCGA | 0.391 | low |
| TCGA-23-1024 | TCGA | 0.851 | high |
| TCGA-04-1361 | TCGA | 1.102 | high |
| TCGA-61-2102 | TCGA | 0.785 | high |
| TCGA-13-0923 | TCGA | 0.830 | high |
| TCGA-29-1697 | TCGA | 0.550 | low |
| TCGA-24-0979 | TCGA | 0.275 | low |
| TCGA-25-2042 | TCGA | 0.702 | high |
| TCGA-25-1627 | TCGA | 0.675 | high |
| TCGA-30-1862 | TCGA | 0.318 | low |
| TCGA-25-1313 | TCGA | 0.641 | low |
| TCGA-13-0795 | TCGA | 0.561 | low |
| TCGA-61-2000 | TCGA | 0.615 | low |
| TCGA-24-2267 | TCGA | 1.377 | high |
| TCGA-13-0884 | TCGA | 0.121 | low |
| TCGA-25-1635 | TCGA | 0.719 | high |
| TCGA-23-1116 | TCGA | 0.762 | high |
| TCGA-13-0893 | TCGA | -0.269 | low |
| TCGA-13-0714 | TCGA | 1.008 | high |
| TCGA-24-2288 | TCGA | 1.241 | high |
| TCGA-31-1956 | TCGA | 1.420 | high |
| TCGA-24-2023 | TCGA | 0.871 | high |
| TCGA-25-1628 | TCGA | 0.953 | high |
| TCGA-04-1519 | TCGA | 0.446 | low |
| TCGA-25-1877 | TCGA | 0.301 | low |
| TCGA-13-1405 | TCGA | 0.683 | high |
| TCGA-23-1123 | TCGA | 1.295 | high |
| TCGA-36-1577 | TCGA | -0.145 | low |
| TCGA-10-0936 | TCGA | 0.981 | high |
| TCGA-04-1350 | TCGA | 0.035 | low |
| TCGA-09-2053 | TCGA | 0.710 | high |
| TCGA-30-1891 | TCGA | 0.377 | low |
| TCGA-24-1544 | TCGA | 0.264 | low |
| TCGA-25-2399 | TCGA | 1.021 | high |
| TCGA-61-1900 | TCGA | 0.592 | low |
| TCGA-24-1846 | TCGA | 0.666 | high |
| TCGA-13-0765 | TCGA | 0.305 | low |
| TCGA-09-1667 | TCGA | 1.232 | high |
| TCGA-29-1766 | TCGA | 0.060 | low |
| TCGA-24-1434 | TCGA | 0.221 | low |
| TCGA-29-1770 | TCGA | 0.120 | low |
| TCGA-20-1686 | TCGA | 0.354 | low |
| TCGA-25-2398 | TCGA | 0.970 | high |
| TCGA-24-2297 | TCGA | 0.158 | low |
| TCGA-29-1705 | TCGA | 1.017 | high |
| TCGA-30-1892 | TCGA | 0.435 | low |
| TCGA-WR-A838 | TCGA | -0.269 | low |
| TCGA-04-1365 | TCGA | 1.197 | high |
| TCGA-24-2024 | TCGA | 0.952 | high |
| TCGA-25-1630 | TCGA | 1.151 | high |
| TCGA-09-1673 | TCGA | 0.551 | low |
| TCGA-57-1586 | TCGA | 0.447 | low |
| TCGA-09-2054 | TCGA | 0.785 | high |
| TCGA-13-1506 | TCGA | 0.745 | high |
| TCGA-29-2427 | TCGA | 1.501 | high |
| TCGA-30-1860 | TCGA | 0.990 | high |
| TCGA-61-2002 | TCGA | 1.556 | high |
| TCGA-24-2289 | TCGA | 0.407 | low |
| TCGA-61-1728 | TCGA | 1.194 | high |
| TCGA-13-0726 | TCGA | 0.524 | low |
| TCGA-13-1477 | TCGA | 0.004 | low |
| TCGA-13-0724 | TCGA | -0.292 | low |
| TCGA-61-1907 | TCGA | 1.412 | high |
| TCGA-13-1483 | TCGA | 0.044 | low |
| TCGA-10-0933 | TCGA | 0.157 | low |
| TCGA-29-2425 | TCGA | 1.205 | high |
| TCGA-36-1570 | TCGA | 0.838 | high |
| TCGA-24-1843 | TCGA | 1.107 | high |
| TCGA-61-1910 | TCGA | 1.034 | high |
| TCGA-25-2392 | TCGA | 1.420 | high |
| TCGA-09-0369 | TCGA | 0.784 | high |
| TCGA-57-1584 | TCGA | 0.952 | high |
| TCGA-09-2056 | TCGA | 1.253 | high |
| TCGA-25-1870 | TCGA | 0.436 | low |
| TCGA-61-1737 | TCGA | 0.978 | high |
| TCGA-09-1665 | TCGA | 0.345 | low |
| TCGA-31-1953 | TCGA | 1.412 | high |
| TCGA-29-1784 | TCGA | 1.412 | high |
| TCGA-29-1710 | TCGA | 1.422 | high |
| TCGA-24-2026 | TCGA | 1.073 | high |
| TCGA-24-1103 | TCGA | 0.645 | low |
| TCGA-59-2352 | TCGA | 0.794 | high |
| TCGA-61-2008 | TCGA | 1.041 | high |
| TCGA-23-1113 | TCGA | 0.470 | low |
| TCGA-24-2261 | TCGA | 1.241 | high |
| TCGA-24-2281 | TCGA | 1.008 | high |
| TCGA-29-1707 | TCGA | 0.891 | high |
| TCGA-29-1691 | TCGA | 1.368 | high |
| TCGA-04-1530 | TCGA | 0.428 | low |
| TCGA-61-1721 | TCGA | 0.867 | high |
| TCGA-24-1546 | TCGA | 0.239 | low |
| TCGA-61-2110 | TCGA | 0.546 | low |
| TCGA-24-2262 | TCGA | 0.679 | high |
| TCGA-13-1407 | TCGA | 0.020 | low |
| TCGA-24-0970 | TCGA | -0.201 | low |
| TCGA-59-2351 | TCGA | 1.034 | high |
| TCGA-24-1844 | TCGA | 0.800 | high |
| TCGA-57-1583 | TCGA | 0.534 | low |
| TCGA-24-0966 | TCGA | 1.244 | high |
| TCGA-30-1718 | TCGA | 0.163 | low |
| TCGA-13-1410 | TCGA | 0.871 | high |
| TCGA-61-2012 | TCGA | 1.054 | high |
| TCGA-61-2009 | TCGA | 0.614 | low |
| TCGA-30-1857 | TCGA | 0.890 | high |
| TCGA-20-1683 | TCGA | 0.700 | high |
| TCGA-04-1341 | TCGA | -0.078 | low |
| TCGA-29-1763 | TCGA | 0.656 | low |
| TCGA-29-1783 | TCGA | 1.034 | high |
| TCGA-23-1809 | TCGA | 1.028 | high |
| TCGA-29-1777 | TCGA | 0.692 | high |
| TCGA-24-1104 | TCGA | 1.151 | high |
| TCGA-24-1469 | TCGA | 1.191 | high |
| TCGA-24-2290 | TCGA | 0.964 | high |
| TCGA-25-2391 | TCGA | 0.883 | high |
| TCGA-23-1114 | TCGA | 0.536 | low |
| GSM249730 | GEO | 0.411 | low |
| GSM249876 | GEO | 0.519 | low |
| GSM249775 | GEO | 0.356 | low |
| GSM249746 | GEO | 0.175 | low |
| GSM249766 | GEO | 0.540 | low |
| GSM249839 | GEO | 0.106 | low |
| GSM249930 | GEO | 0.929 | high |
| GSM249822 | GEO | 0.869 | high |
| GSM249987 | GEO | -0.199 | low |
| GSM249975 | GEO | 0.485 | low |
| GSM249894 | GEO | 1.095 | high |
| GSM249821 | GEO | 0.108 | low |
| GSM249883 | GEO | 0.975 | high |
| GSM249758 | GEO | 0.206 | low |
| GSM249913 | GEO | 0.726 | high |
| GSM249940 | GEO | 0.603 | low |
| GSM249824 | GEO | -0.140 | low |
| GSM249960 | GEO | 0.961 | high |
| GSM249936 | GEO | 0.149 | low |
| GSM249927 | GEO | 0.147 | low |
| GSM249778 | GEO | 0.819 | high |
| GSM249959 | GEO | -0.047 | low |
| GSM249881 | GEO | 0.707 | high |
| GSM249850 | GEO | 0.236 | low |
| GSM249760 | GEO | 0.870 | high |
| GSM249835 | GEO | 0.398 | low |
| GSM249740 | GEO | -0.012 | low |
| GSM249958 | GEO | 0.489 | low |
| GSM249714 | GEO | -0.056 | low |
| GSM249727 | GEO | -0.010 | low |
| GSM249869 | GEO | -0.229 | low |
| GSM249736 | GEO | 0.994 | high |
| GSM249759 | GEO | 0.238 | low |
| GSM249905 | GEO | 0.220 | low |
| GSM249827 | GEO | 0.356 | low |
| GSM249749 | GEO | 0.918 | high |
| GSM249769 | GEO | 0.414 | low |
| GSM249770 | GEO | 0.287 | low |
| GSM249992 | GEO | 0.094 | low |
| GSM249981 | GEO | 0.772 | high |
| GSM249968 | GEO | 1.320 | high |
| GSM249735 | GEO | 0.514 | low |
| GSM249750 | GEO | 1.408 | high |
| GSM249723 | GEO | 0.129 | low |
| GSM249792 | GEO | 0.718 | high |
| GSM249805 | GEO | -0.115 | low |
| GSM249969 | GEO | 0.042 | low |
| GSM249724 | GEO | -0.039 | low |
| GSM249717 | GEO | 0.194 | low |
| GSM249950 | GEO | -0.099 | low |
| GSM249721 | GEO | 0.448 | low |
| GSM249783 | GEO | 0.212 | low |
| GSM249939 | GEO | 0.759 | high |
| GSM249956 | GEO | 1.149 | high |
| GSM249793 | GEO | 0.909 | high |
| GSM249722 | GEO | 0.042 | low |
| GSM249809 | GEO | 0.201 | low |
| GSM249784 | GEO | 0.165 | low |
| GSM249965 | GEO | 0.687 | high |
| GSM249976 | GEO | 0.381 | low |
| GSM249738 | GEO | 0.407 | low |
| GSM249739 | GEO | 0.355 | low |
| GSM249866 | GEO | 0.175 | low |
| GSM249756 | GEO | 0.383 | low |
| GSM249900 | GEO | 0.772 | high |
| GSM249812 | GEO | 0.125 | low |
| GSM249983 | GEO | 0.716 | high |
| GSM249994 | GEO | 0.811 | high |
| GSM249875 | GEO | -0.281 | low |
| GSM249808 | GEO | 1.383 | high |
| GSM249776 | GEO | 0.184 | low |
| GSM249897 | GEO | 0.918 | high |
| GSM249811 | GEO | -0.021 | low |
| GSM249993 | GEO | 1.164 | high |
| GSM249855 | GEO | 0.892 | high |
| GSM249830 | GEO | 0.189 | low |
| GSM249745 | GEO | 0.490 | low |
| GSM250000 | GEO | 0.748 | high |
| GSM249732 | GEO | -0.236 | low |
| GSM249854 | GEO | 0.568 | low |
| GSM249819 | GEO | 0.251 | low |
| GSM249744 | GEO | 0.196 | low |
| GSM249728 | GEO | 0.499 | low |
| GSM249763 | GEO | 0.685 | high |
| GSM249743 | GEO | -0.011 | low |
| GSM249731 | GEO | 0.194 | low |
| GSM249874 | GEO | 1.350 | high |
| GSM249929 | GEO | 0.347 | low |
| GSM249957 | GEO | -0.034 | low |
| GSM249777 | GEO | 0.747 | high |
| GSM249928 | GEO | 0.929 | high |
| GSM249932 | GEO | -0.150 | low |
| GSM249820 | GEO | 0.862 | high |
| GSM249785 | GEO | 0.236 | low |
| GSM249910 | GEO | 0.562 | low |
| GSM249847 | GEO | -0.257 | low |
| GSM249802 | GEO | 0.081 | low |
| GSM249729 | GEO | 0.626 | low |
| GSM249943 | GEO | 0.090 | low |
| GSM249988 | GEO | -0.034 | low |
| GSM249799 | GEO | -0.005 | low |
| GSM249934 | GEO | 0.233 | low |
| GSM249916 | GEO | 0.050 | low |
| GSM249880 | GEO | 1.178 | high |
| GSM249725 | GEO | 0.550 | low |
| GSM249942 | GEO | 0.175 | low |
| GSM249907 | GEO | -0.001 | low |
| GSM249962 | GEO | 0.694 | high |
| GSM249789 | GEO | 0.417 | low |
| GSM249826 | GEO | 0.043 | low |
| GSM249961 | GEO | 0.879 | high |
| GSM249941 | GEO | 0.078 | low |
| GSM249933 | GEO | 1.298 | high |
| GSM249716 | GEO | 0.472 | low |
| GSM249734 | GEO | -0.011 | low |
| GSM249852 | GEO | 1.234 | high |
| GSM249762 | GEO | 0.996 | high |
| GSM249871 | GEO | 0.595 | low |
| GSM249742 | GEO | -0.056 | low |
| GSM249788 | GEO | 0.817 | high |
| GSM249999 | GEO | 0.342 | low |
| GSM249851 | GEO | 0.459 | low |
| GSM249741 | GEO | 0.229 | low |
| GSM249733 | GEO | -0.011 | low |
| GSM249815 | GEO | 0.460 | low |
| GSM249715 | GEO | 0.658 | low |
| GSM249926 | GEO | 0.865 | high |
| GSM249898 | GEO | 0.204 | low |
| GSM249904 | GEO | 0.854 | high |
| GSM249833 | GEO | -0.019 | low |
| GSM249841 | GEO | 0.080 | low |
| GSM249990 | GEO | -0.202 | low |
| GSM249751 | GEO | 1.062 | high |
| GSM249842 | GEO | 0.883 | high |
| GSM249752 | GEO | 0.189 | low |
| GSM249972 | GEO | -0.036 | low |
| GSM249951 | GEO | 0.113 | low |
| GSM249726 | GEO | 0.535 | low |
| GSM249889 | GEO | 1.111 | high |
| GSM249737 | GEO | 0.022 | low |
| GSM249971 | GEO | 0.410 | low |
| GSM249825 | GEO | 0.406 | low |
| GSM249780 | GEO | 0.346 | low |
| GSM249899 | GEO | -0.152 | low |
| GSM249953 | GEO | 0.782 | high |
| GSM249718 | GEO | 0.320 | low |
| GSM249857 | GEO | 0.485 | low |
| GSM249747 | GEO | 0.280 | low |
| GSM249767 | GEO | 0.261 | low |
| GSM249720 | GEO | 0.746 | high |
| GSM249919 | GEO | -0.076 | low |
| GSM249973 | GEO | 0.726 | high |
| GSM249954 | GEO | 1.062 | high |
| GSM249796 | GEO | 0.319 | low |
| GSM249877 | GEO | 0.330 | low |
| GSM249967 | GEO | -0.136 | low |
| GSM249986 | GEO | 0.291 | low |
| GSM249902 | GEO | 0.395 | low |
| GSM249774 | GEO | -0.019 | low |
| GSM249753 | GEO | 0.189 | low |
| GSM249918 | GEO | 0.486 | low |
| GSM249996 | GEO | 0.496 | low |
| GSM249920 | GEO | 0.003 | low |
| GSM249719 | GEO | 0.393 | low |
| GSM249844 | GEO | 0.345 | low |
| GSM249754 | GEO | 0.615 | low |
| GSM249832 | GEO | 0.432 | low |
| GSM249773 | GEO | 0.062 | low |
